# Patient triage system for supporting the operation of dispatch centres and rescue teams

**DOI:** 10.1186/s12911-021-01440-x

**Published:** 2021-02-19

**Authors:** Acrapol Nimmolrat, Krongkarn Sutham, Orawit Thinnukool

**Affiliations:** 1 College of Arts, Media and Technology, Chiang Mai University, Chiang Mai, 50200 Thailand; 2 Department of Emergency Medicine, Faculty of Medicine, Chiang Mai University, Chiang Mai, 50200 Thailand; 3 Research Group of Embedded Systems and Mobile Application in Health Science, College of Arts, Media and Technology, Chiang Mai University, Chiang Mai, 50200 Thailand

**Keywords:** Triage, Emergency medical services, Mobile application, Dispatch centre, Emergency development system

## Abstract

**Background:**

The Thai medical application for patient triage, namely Triagist, is an mHealth application designed to support the pre-hospital process. However, since the functions of the application that are necessary for the pre-hospital process have been found not to be fully developed, the addition of a back-end system has been considered to increase its performance and usability.

**Objective:**

To determine the ability of the previous version to effectively manage the pre-hospital process and analyse the current problems with the pre-hospital operation. Therefore, the new system was developed to support the connection of dispatch centres or operational centres to the Triagist mobile application and system evaluation.

**Method:**

Design thinking methodology was used to analyse, design and develop a patient triage system to support the pre-hospital process in Thailand based on users’ requirements. 68 active members of the rescue teams and emergency medical staff in Chiang Mai and Lampang provinces were recruited to test the reliability of the system based on a prototype application.

**Results:**

The new medical mobile application for patient triage in Thailand was validated for use due to containing the two essential functions of Initial Dispatch Code (IDC) geolocation and IDC management. When the system was tested by emergency staff who were responsible for using it, those with the least experience were found to use it better than their highly experienced colleagues. Moreover, in cases where the system had been implemented, it was found to determine the frequency of symptoms, the time period during which cases occurred, and the density of cases in each area.

**Conclusion:**

This system, which has been developed based on the use of smart technology, will play an important role in supporting emergency services in Thailand by enhancing the efficiency of the pre-hospital process. Emergency centres will receive IDC information from the geolocation system so that they can determine patients’ location without undue delay. Emergency services will be able to rapidly prepare the necessary resources and administrative tasks will be supported by linking the dispatch centre to central rescue teams.

## Background

Mobile healthcare applications have been applied in a variety of medical settings, especially in this era of digital technology, and an increasing number of people are using smart technology to access primary healthcare via a smart phone [1]**.** The advanced development of smart technology has led to it being widely used for healthcare purposes, for example, monitoring patients [2–4], consulting experts or doctors [5–7], healthcare medication and diagnosis [8], and health education [9]. The most common healthcare services provided via a mobile application are reported to be primary care (41%) and prevention (47%) [10].

Healthcare applications based on smart technology have become extremely popular in emergency medical settings in several countries. Many systems have been developed to enable first responders or emergency staff to rapidly assess patients’ condition in order to triage them at each stage of healthcare management [11–14] because triage is the key to effective emergency medicine management [15]. The triage in healthcare management consists of three stages, namely, pre-hospital triage (stage 1), which involves dispatching an ambulance and pre-hospital care resources, triage at the scene (stage 2) by first response emergency staff who attend the patient, and triage on arrival at the hospital or emergency unit (stage 3) [16]. Several smart systems have been widely used in order to reduce the time consumed in the triage process based on supporting medical staff’s rapid decision-making [17] and patients’ self-triage [18].

Thailand’s emergency system [19] is based on the Anglo-American Model (AAM) combined with Criteria Based Dispatch (CBD), which is focused on the pre-hospital process. This method involves the use of five colour codes to rapidly identify the condition of patients who are requesting treatment before admitting them to a hospital. The Initial Dispatch Codes (IDCs) are classified in Table 1, together with an explanation of each of their essential response.

**Table 1:** Explanation of initial dispatch Code (IDCs)

Colour	Triage criteria	Essential response
Red	Critical emergency patients	The Basic Life Support Unit (BLS) responds to the patient *within 4 min* of the accident and the Advanced Life Support unit (ALS) responds *within 8 min* of the accident
Yellow	Urgent emergency patients	The BLS responds to the patient *within 8 min* of the accident and the First Response Unit (FR) responds within 15 min of the accident
Green	Non-urgent emergency patients	The BLS responds to the patient *within 8 min* of the accident
White	General patients	Response to patient is via telephone referral programme and consideration of BLS
Black	Not patients	No response needed

According to a report published after the Thailand Academic Conference on Emergency Medicine in 2019, the next generation of Thai emergency medical services (EMS) must be based on a digital platform [20]. Therefore, the National Institute for Emergency Medicine of Thailand (NIEM) has already designed and developed several systems to support the country’s EMS divided into the 25 main categories of symptoms [21, 22], as shown in Table 2.

**Table 2:** 25 main symptoms according to the National Institute for Emergency Medicine (NIEM)

Code	Symptom/condition	Code	Symptom/condition
1	Abdominal/Back/Groin Pain	14	O.D./Poisoning
2	Anaphylaxis/Allergic Reaction	15	Pregnancy/Childbirth/Gyn
3	Infectious Disease	16	Seizures
4	Bleeding (Non-traumatic)	17	Sick (Unknown)/Other
5	Breathing Difficulty	18	Stroke (CVA)
6	Cardiac Arrest	19	Unconscious/Unresponsive/ Syncope
7	Chest Pain/Discomfort/Heart Problems	20	Pediatric Emergencies
8	Choking	21	Assault/Trauma
9	Diabetic	22	Burns—Thermal/Electrical/Chemical
10	Environmental/Toxic Exposure	23	Drowning/Near Drowning/Diving or Water-related Injury
11	Undefined symptoms	24	Falls/Accidents/Pain
12	Head/Neck	25	Motor Vehicle Accident (MVA)
13	Mental/Emotional/Psychological	–	–

However, the main problem with developing healthcare applications based on smart technology is the lack of Thailand’s information technology infrastructure compared to other countries [23–25]. For instance, the associated system to triage patients developed by the NIEM did not work in practice; therefore, every Thai medical institution has been encouraged to develop its own smart technology to resolve this problem.

### Prior work

Sutham and her team [14] recently developed a Thai mobile application to triage patients in support of Thailand Science Research and Innovation (TSRI), using the emergency Severity Index (ESI) as the standard of classification in the application. This system was suitable for rescue teams, community hospitals and emergency medical volunteers to triage patients.

When this application was implemented, it was found to identify the IDC, which led to the allocation of emergency resources. When patients make a phone call to a dispatch centre or local emergency centre, the IDC can identify the level of urgency and medical resources needed, while simultaneously sending data about their condition to the dispatch centre via an email, SMS or Line application.

However, some weaknesses were highlighted by the pilot test; for instance, the application did not completely connect to the dispatch centre, did not respond to geolocation data, the triage results were not recorded in an appropriate format and it was hard to recheck information about emergency cases.

Therefore, in order to further enhance the management of emergencies by dispatch centres or rescue team centres based on the use of ordinary channels such as emergency phone calls and the Triagist mobile application, it is necessary to consider the functions of the previous version to assess its ability to effectively manage the first stage of emergency medicine. Therefore, the purpose of this research is to design and develop a new system to support the connection of the Triagist mobile application to dispatch centres or operational centres. This will be a web-based platform that has the ability to recheck the IDC results and identify the geolocation of services by referring to several current researchers [26–28], who have also developed a new system.

### Implementation of related emergency systems

Several researchers have developed triage systems that contain the criteria necessary to classify patients’ condition. Some of the research and development associated with emergency medicine is detailed below.

Sutham et al. [14] recently developed a triage medical application called Triagist, which can be used to classify the level of emergency based on an IDC. The Triagist can be used as a practical tool, as well as an educational one, for new emergency staff who lack comprehensive emergency medical knowledge and skills. This computerised triage system corresponds to the primary emergency medicine stage.

Sumrumeram [29] developed a medical application that can track high-risk stroke and stemi patients who require time-sensitive EMS. This application is based on smartphone technology and a Global Positioning System (GPS) that can track patients, accurately pinpoint their location and classify their status. Hence, it is of great benefit to both the EMS and patients by optimising the survival rate. This computerised triage system corresponds to the primary stage of emergency medicine.

Weinlich et al. [27] applied the geolocation from a smartphone to enable the EMS to quickly respond to emergency calls. When testing the accuracy of the GPS, Wi-Fi (wireless LAN network) and LBS (location-based system) in eleven different countries, the combination of Wi-Fi geolocation and GPS was found to accurately identify emergency locations, especially within or close to buildings.

Stein et al. [30] designed and developed a triage system for use in dental emergencies. This system can identify tooth symbols based on intraoral images, which helps dentists to assess the condition of a tooth. Moreover, users can self-identify the condition of the tooth before requesting dental services. This system is an example of the primary stage of emergency medicine.

Romano et al. [31] used smart technology in the form of a mobile phone to support emergency responders. The geolocation and necessary information of the patient, such as photos or videos, are delivered directly to the operational centre. This is a practical system that facilitates a faster response, thereby demonstrating the ability of the geolocation to help EMS to locate the patient prior to the pre-hospital process.

Wallis et al. [32] researched and developed a smartphone application that burn injury patients can use for consultation in an emergency. This application enables users to indicate the specific injured bodily surface(s) and calculate the total bodily surface area of the burn and automatically send a text message to the experts, who can quickly respond and help the patients to triage themselves prior to the pre-hospital process. This system is another example of basic triage at the primary stage of emergency medicine.

## Method

### Participants

The sample of the current study consisted of 68 volunteers (n = 68) from rescue teams active in Chiang Mai and Lampang, as well as emergency medical staff. They were recruited using a purposive sampling technique. The samples were classified into three groups of rescue teams, volunteers, and emergency medical staff based on at least two years of emergency operation experience. Each group was divided into five sub-groups according to their experience of operating in emergency situations and their demographics are shown in Table 3.

**Table 3:** Demographics of samples (n = 68)

Points	Characteristics	Percentage	(n)
Gender	Male	73.53	50
	Female	26.47	18
Experience A:	2–3 Years	39.71	27
B:	3–4 Years	32.35	22
C:	5–6 Years	11.76	8
D:	7–8 Years	11.76	8
E:	9–10 Years	4.41	3
Emergency type	Rescue teams	55.88	38
	Volunteers	26.47	18
	Emergency Medical Staff	17.65	12

### Software development process

The development of the software for dispatch centres or rescue team centres was designed to solve the problems in relation to the pre-hospital process. Design thinking was used as the methodology to develop a functional system based on understanding users’ needs, challenging assumptions, redefining problems and creating innovative solutions to test a prototype [33, 34]. This method consisted of the 5 stages detailed below.

*Stage 1 Empathise* This stage involved collecting and analysing data from volunteers to determine the system requirements, such as the functions necessary for dispatch centre or rescue teams to triage patients. Four open-ended questions were sent via a documentary and Google format to emergency doctors, dispatch centre staff and rescue teams.

*Stage 2 Define* The necessary factors for developing the web-based system in terms of functionality, convenience and accessibility were considered at this stage and the back-end functional requirements were identified according to the software requirements from the previous stage.

*Stage 3 Ideate* This stage involved designing the functions, graphical user interface and system architecture. The functions were designed by considering the practical use in a real operation by contacting users via a phone call together with the Triagist application and the system was designed based on the requirements of the user interface. The components of the front-end system design included a menu list, geolocation graphic, navigation and input controls. The Eight Golden Rules of interface design and Nielsen’s Ten Heuristics were used to design an appropriate graphical interface [35, 36].

*Stage 4 Prototype* The prototype was produced by the Laravel programme using the findings from the earlier stages to obtain the best possible functionality to support the provincial dispatch centres [37]. This highly-regarded software platform was chosen to develop the system for the dispatch centres or rescue team centres due to its provision of strong configuration and high technical standard. This platform also enables the use of a universal extendable dashboard, inspector, reusable components, authentication, authorisation, and the integration of tools to accelerate web applications [38]. This framework is suitable for modification to connect the Triagist application and patient triage system. The triage information obtained from the application is recorded in the Firebase of the web-based system.

*Stage 5 Test *This stage included testing the system based on a usability test to ensure the quality and reliability of the back-end and front-end design. 68 active members of the rescue teams in Chiang Mai and Lampang, as well as emergency medical staff, were provided with an indirect link to download and test the preliminary application.

Having established a scenario to test the system, the participants were sent a system manual, username and password to log into the system via email. They were asked to complete the tasks by.

1. Logging into the system using a tablet or personal computer.
2. Finding the IDC results from any report.
3. Finding the patient’s condition.
4. Understanding the patient’s condition.
5. Identifying and approving the geolocation using the Triagist mobile application.
6. Approving the IDC case.

Each participant was randomly assigned three IDCs and, after completing the allotted tasks, they were required to give feedback in an online questionnaire in order to evaluate users’ satisfaction with the system.

The next critical process for the success of novel smart health technology is a usability test, which can be conducted in a number of ways. One of these is to issue a usability questionnaire to collect feedback from target users [39, 40]. Lewis [41] proposed that an after-scenario questionnaire based on the same target users who identified the system requirements would constitute a critical test. The questions for post-task ratings were developed from a Computer System Usability Questionnaire (CSUQ).

This step was required to ensure the quality and reliability of the back-end and front-end design of the system based on 17 criteria to determine the quality of the software. The participants in this study were also required to complete a questionnaire at the end of the scenario trial, but the rating scales of the evaluation in the after-scenario questionnaire were redesigned to contain 5 scales. The usability test criteria and descriptions are shown in Table 4.

**Table 4:** Usability test criteria [41]

Points to consider
1. This system was easy to use
2. I could effectively complete tasks and scenarios using this system
3. I could complete tasks and scenarios quickly using this system
4. I was able to efficiently complete tasks and scenarios using this system
5. I felt comfortable using this system
6. It was easy to learn to use this system
7. I believe I could become productive quickly using this system
8. The system gave error messages that clearly told me how to fix problems
9. I could recover easily and quickly if I made a mistake using the system
10. The information (such as online help, on-screen messages, and other documentation) provided in this system was clear
11. It was easy to find the information I needed
12. The information provided in the system was easy to understand
13. The information was effective in helping me complete tasks and scenarios
14. The information on the system screen was clearly organised
15. This system has a pleasant interface
16. This system has the functions and capabilities I expected it to have

The results were evaluated as a fixed point. The Triagist application was incorporated with the patient triage system for implementation after it had been validated.

## Results

The development of the software for the dispatch centres or rescue team centres was designed to solve problems associated with the pre-hospital process. The software development process consisted of 5 stages and the results of each stage are shown below.

### Results of empathise stage

The results of interviewing 45 respondents were summarised and shown in Table 5. This information, which was collected from emergency doctors, dispatch centre staff and rescue teams, was used to determine the system requirements.

**Table 5:** Results of interviewees using open-ended questions based on the most frequent answers (cut off at 10).

Questions	Group answers/most frequency	Frequency of answers
1. What do you want from the Triagist application after the trial?	Screen symptoms and alert medical services	40
	Automatically require the services of EMS	35
	Support medical emergency	28
	Link information to an emergency area	22
	Link to government system or sent data to an emergency center	11
2. What makes it difficult for you to manage an emergency service?	Difficult to assess the emergency from a phone call	33
	Misunderstanding of cases reported by telephone	31
	Difficult to obtain information from relatives and patients	28
	Hard to track patient information (condition and primary report)	27
	Hard to find the location of the patient from rescue information	26
	Skills of emergency staff	22
	System’s connection to emergency centre or rescue unit	15
	Emergency policy	10
3. Have you any experience of smart technology in emergency medicine? If so, what kind of system or procedure?	Radio/phone call	40
	Hotline EMS 1669	36
	Local emergency hotline service	33
	Local hospital hotline service	25
	Application of EMS	24
	Line application	16
	etc	10
4. What would be a good solution from the future services of a Triagist mobile application?	A function that helps to triage patients for primary treatment	41
	A function that connects to a hospital or provincial emergency centre	39
	A function that navigates directly to the emergency location	32
	A function to report the location	29
	A function that automatically records treatment information and sends it to the emergency centre	28
	A function that calls the emergency doctor	21
	A function that links it to the EMS	13
	etc	10

#### Results of define stage

The factors necessary for developing the patient triage system in terms of functionality, convenience and accessibility were considered based on the results of the empathise stage. The functions were considered stepwise, as shown in Table 6.

**Table 6:** Function analysis and design of Patient Triage System for Supporting the Operation of Dispatch Centres and Rescue Teams

Problems/ necessary factors	Fixing solution
Difficult to access the emergency location/ hard to address the patient’s location or emergency area/report location	Provide a function to accurately access the emergency area A function that navigates directly to the emergency location
Difficult to obtain information from relatives and patients or primary information	Provide information of IDC and issue a primary report on patient’s condition to the emergency system
	A function to send or access the patient’s information to identify the IDC to the emergency system A function that records treatment information and automatically sends it to the emergency centre
A function that links to EMS or any service	Design the system to link the Triagist with the emergency system

### Results of ideate stage

The design of the possible functions of the system was based on a range of perspectives from team members’ experience and exposure. The problems with the current operational procedure and use of the Triagist application, which were collected from the interviews, are summarised below along with the possibility of correcting them.

1. Develop a function to access the geolocation of users or patients to facilitate navigation to the emergency site.
2. The system should choose which current system to support (Government or EMS).
3. The system should choose which emergency unit to provide with the IDC information.
4. The system should choose which data of patients or users to provide or retrieve about their’condition to support the allocation of emergency resources.

#### System architecture

**Fig. 1:**
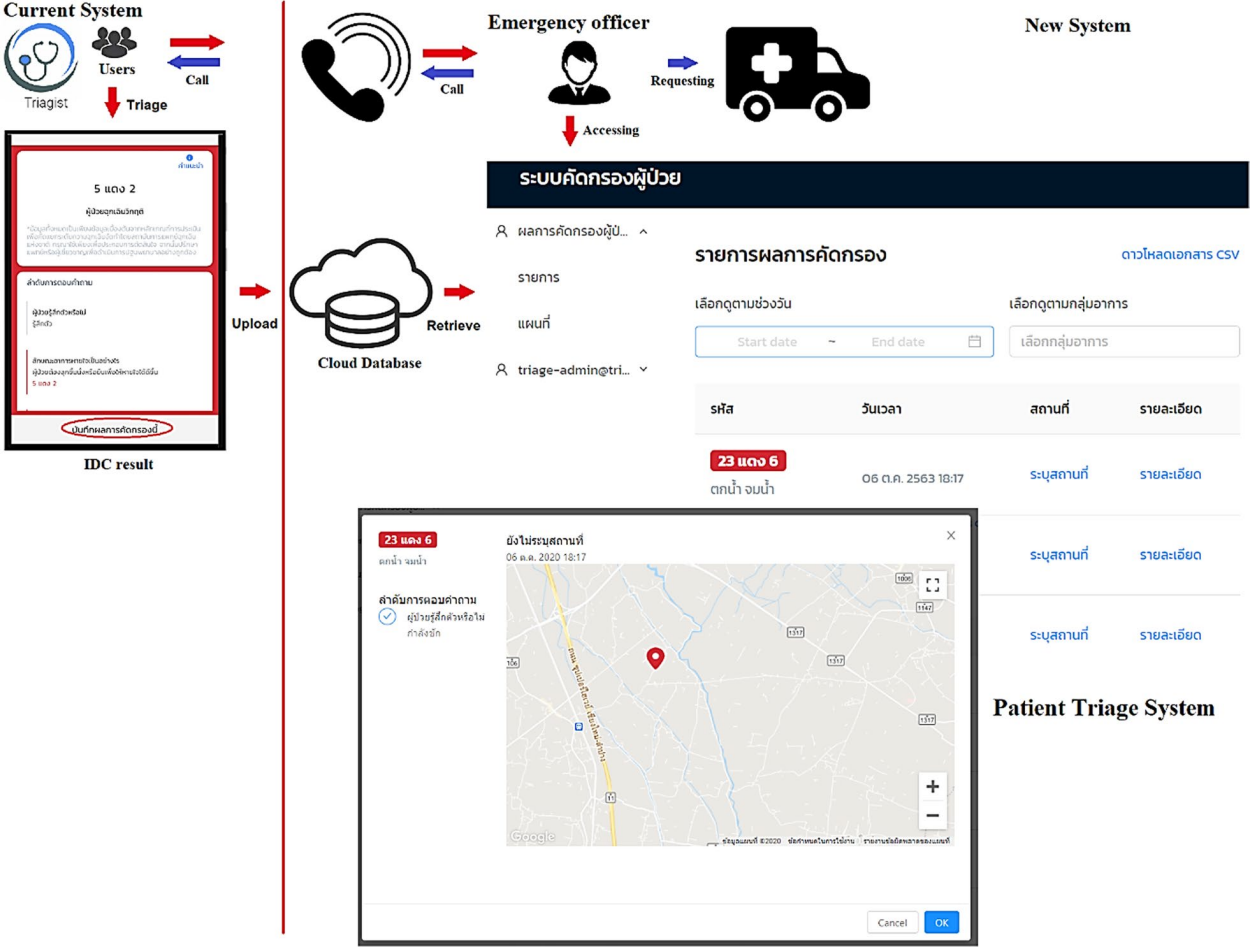
Overview of the patient triage system for dispatch centres or rescue teams. Note: The blue arrow indicates the future operation after the Triagist application is accepted for operation with the current Thai EMS.

Based on the problem analysis from the previous step, the system in Fig. 1 represents the overall possible system in this study, which is a combination of the current Triagist application and a patient triage system to support dispatch centres or rescue teams. This design is based on the possible development of the health promotion law. The Triagist application provides IDC results to indicate patients’ status, whereas the patient triage system can retrieve the IDC results with a list of each patient’s condition, geolocation, and the date and time. In cases of emergency, the application enables emergency officers to assess patients’ IDC results in real time. In critical cases, they can directly call the patient to determine the situation and then request emergency resources via Government EMS, such as an ambulance, medical staff or rescue team to handle the emergency in a timely manner. Moreover, the patient triage system can give medical doctors information about patients’ condition before they arrive at the hospital; hence, it supports three hospital triage systems in healthcare management. However, data privacy is also considered in order to protect patients’ personal data. The Triagist application and the patient triage system both anonymise users’ personal information.

#### System development analysis and design

The necessary functions and graphical user interface for the development of the system were designed by analysing the current patient triage system, which was also designed to support emergency dispatch centres or rescue teams. The results of the analysis of the functionalities of the system are shown in Table 7.

**Table 7:**
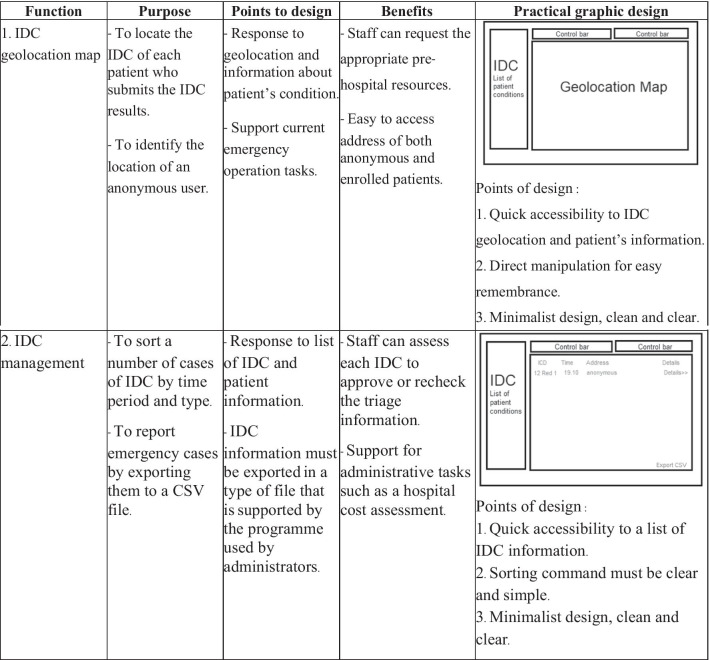
Functionalities of the patient triage system for provincial dispatch centres

### Result of prototyping stage

The result of the prototyping stage was the generation of a software prototype based on the summary of the necessary functions and graphical user interface of the wireframes.

#### Patient triage system interface development

**Fig. 2:**
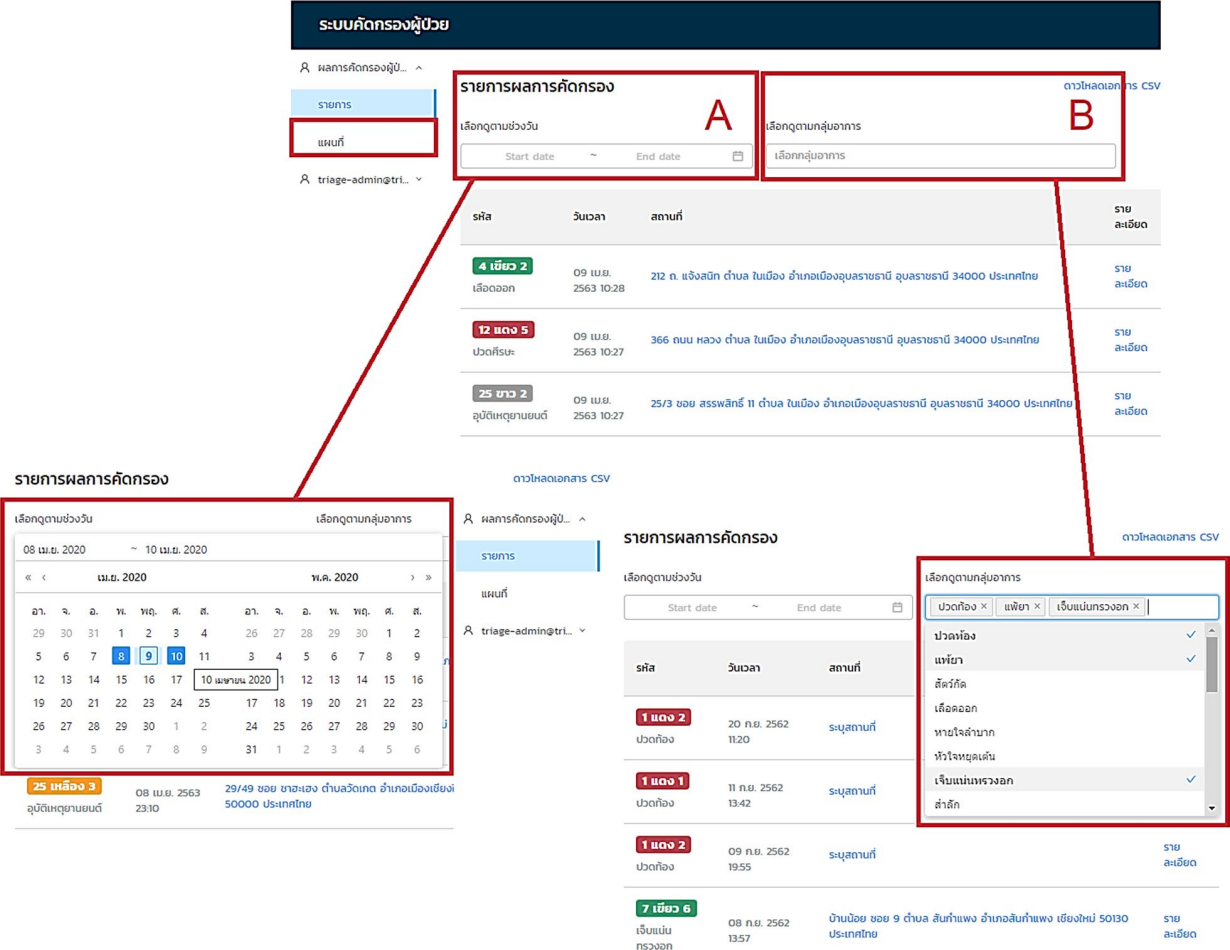
Screenshot of the triage geolocation map. The IDC results classified by start date and end date are shown in rectangle A and by patients’ symptoms in rectangle B

Firstly, the geolocation of the triage medical system was developed. This function, together with the patient’s condition, enables the level of emergency to be evaluated. The IDC results illustrated in the geolocation map are shown in Fig. 2. Each coloured point on the map represents the level of IDC according to Table 2. Moreover, these results can be classified by start date to end date and by patients’ symptoms covering the 25 symptom categories of Sutham et al. [14].

**Fig. 3:**
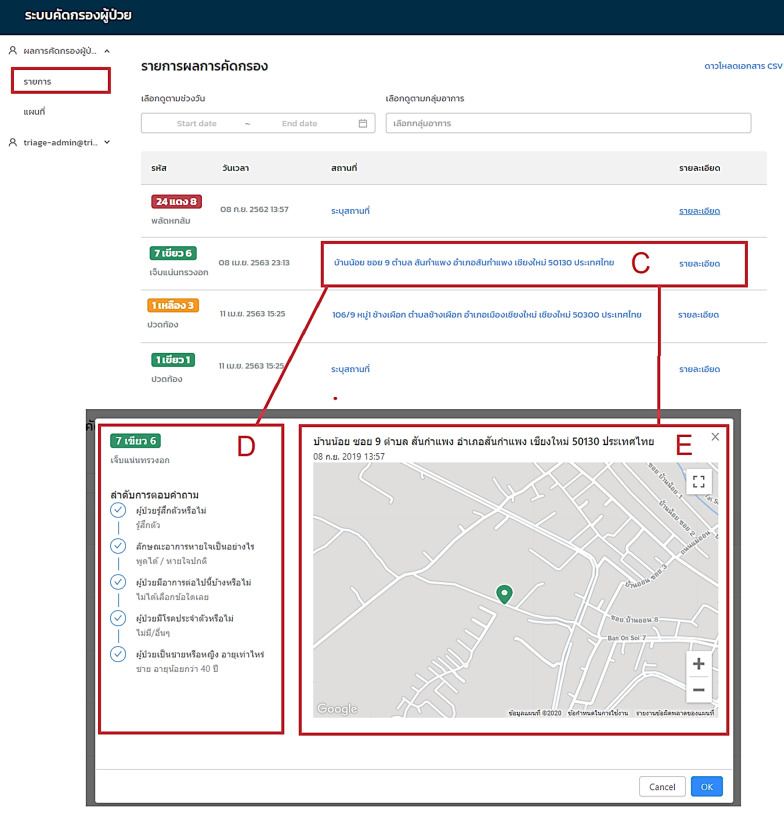
Screenshot of the IDC results list and geolocation information report

The screenshot in Fig. 3 shows the list of IDC results retrieved from the Triagist users. The sequence listing of case submissions is displayed in Rectangle C with the IDC level, date and time, and location. Individual cases in terms of the patient’s habits are displayed in Rectangle D following the CBD questions in the application. Meanwhile, the patient’s location is shown as a detailed location and geolocation map (rectangle E). The Triagist application will be monitored for any missing geolocations by the staff who operate the system.

### Results of testing stage

In the final stage, the prototype was tested. This included testing the system based on a usability test to ensure the quality and reliability of the back-end and front-end design.

#### Results of patient triage system assessment

**Fig. 4:**
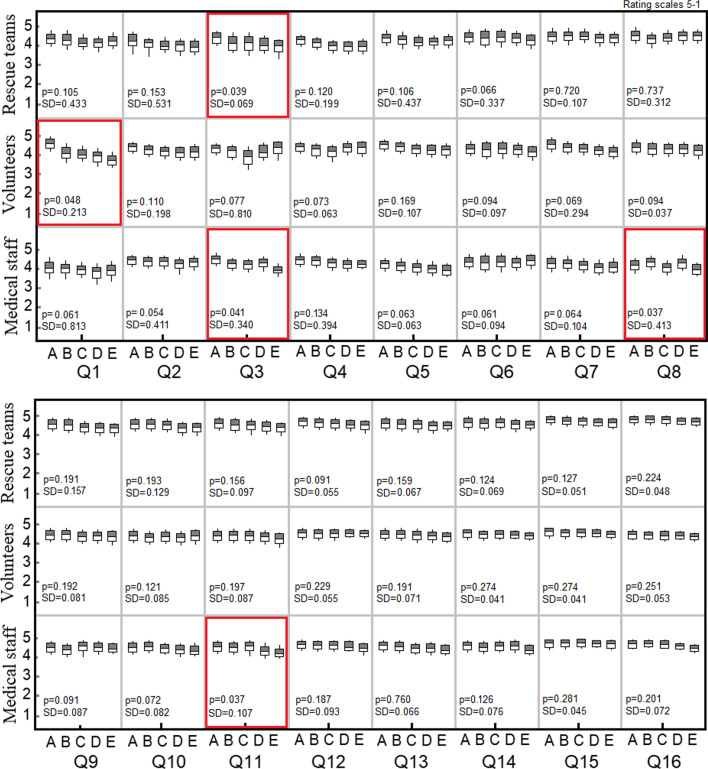
Comparison of the results of users’ feedback that correspond to the usability test criteria [41]. The rating scores are divided into the three groups of medical staff, volunteers and rescue teams. The p-values and standard deviation (S.D.) are presented below the box plots. Labels Q1-Q16 represent each question that corresponds to Table 4. Labels **a**–**e** represent the number of years of the sample group’s emergency experience: **a** 2–3 years, **b** 3–4 years, **c** 5–6 years, **d** 7–8 years, **e** 9–10 years

After testing the scenarios, the three groups of participants were asked to complete a usability questionnaire which corresponded to the usability test criteria in Table 4 [41]. The results are presented in Fig. 4. The average rating score is divided into three groups of rescue teams, volunteers and medical staff based on their experience of emergency operations.

According to the statistical analysis, the majority of the users, especially the rescue teams, who were the target users of the application, indicated that they were satisfied with it. They gave the highest mean score of 4.89 to the statement, “the system has all the necessary functions and capabilities”. When the average scores for the emergency operation experience of each group were analysed, there were almost no statistically significant differences (p < 0.05), apart from the scores in response to questions 1, 3, 8 and 11.

Statistically significant differences were found in the volunteer group’s response to question 1, which was related to the system’s ease of use. Volunteers, who had little experience, gave a more positive average rating than the highly experienced group with a statistical significance of 0.05. Meanwhile, the scores of both the medical staff and rescue teams in response to question 3, which asked if users were able to complete tasks and scenarios quickly using this system, were classified by their emergency operation experience, and it was found that the highly experienced group gave a lower score than the group with little experience. In addition, the answers to Q8 and Q11 showed a similar trend with age range and experience affecting the scores, especially of the medical staff. In considering the interesting points of the significantly different responses of each group to each criterion, it was found that the experience of the group of medical staff and rescue teams usually depended on their age. This was in contrast to the volunteer group, whose ages and experience of emergency operations were varied.

Although there were no significant statistical differences in the scores of the usability evaluation of the other questions (Q2, Q4, Q5, Q6, Q7, Q9, Q10 and Qs12-16) by those respondents who had a great deal of experience of emergency operations, the majority of the ratings showed a similar trend of a higher score by the group with little experience than those in the highly experienced group.

#### Patient triage system test results

**Fig. 5:**
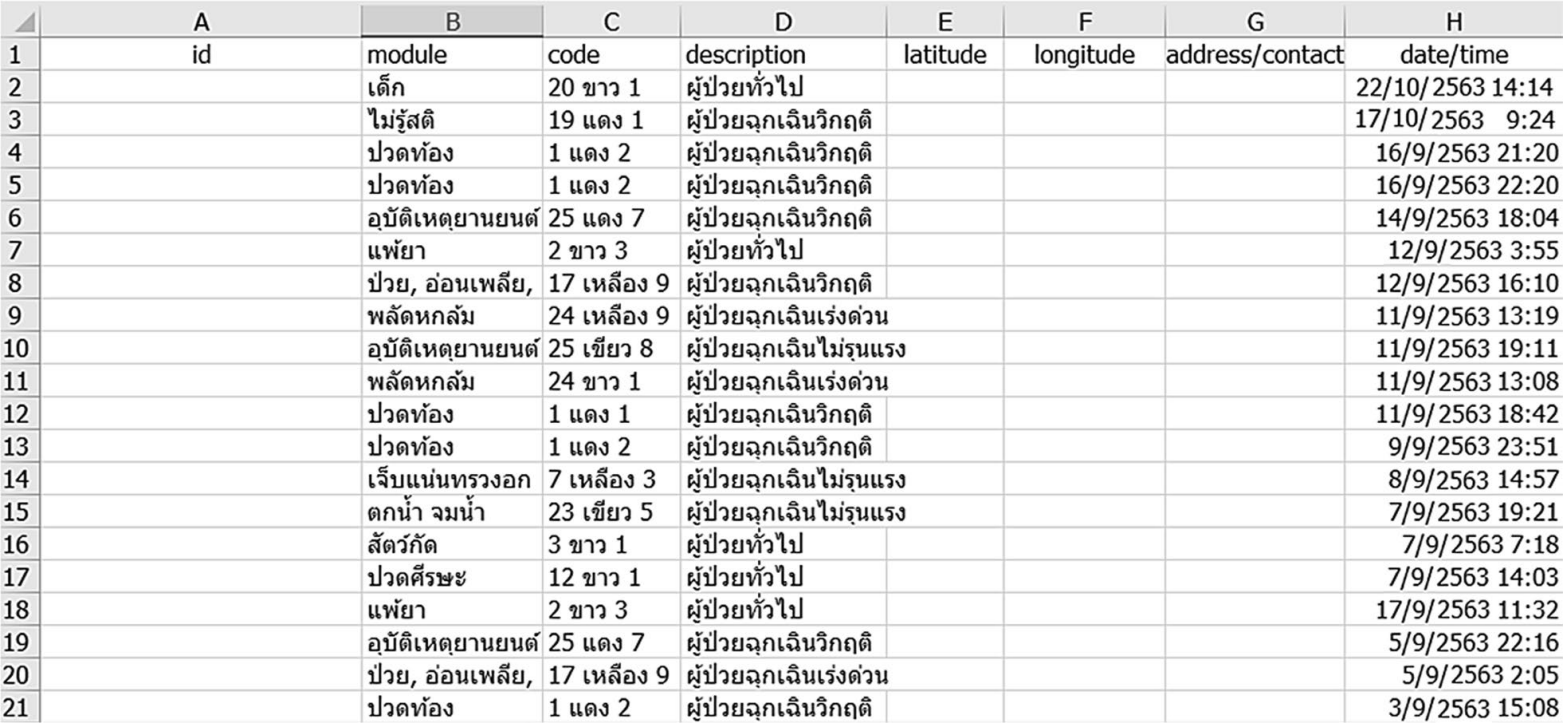
Raw data retrieved from the.CSV file of the patient triage system. Note: some users’ information was omitted from the system to preserve their anonymity

The fixed version of the Triagist mobile application was improved in order to support its connection with the current patient triage system. The validated application was released to the Google Play store and, without being promoted, a total of 178 downloads were reported from the google developer account in September 2019. The IDC results were analysed by collecting the.CSV file from the cloud database of the patient triage system, which contains users’ information, including user ID, symptoms, IDC code, patient status, latitude, longitude, address information and date/time of case, respectively. The raw data from the.CSV file is shown in Fig. 5, although user ID, latitude, longitude and contact address are omitted for privacy purposes.

**Fig. 6:**
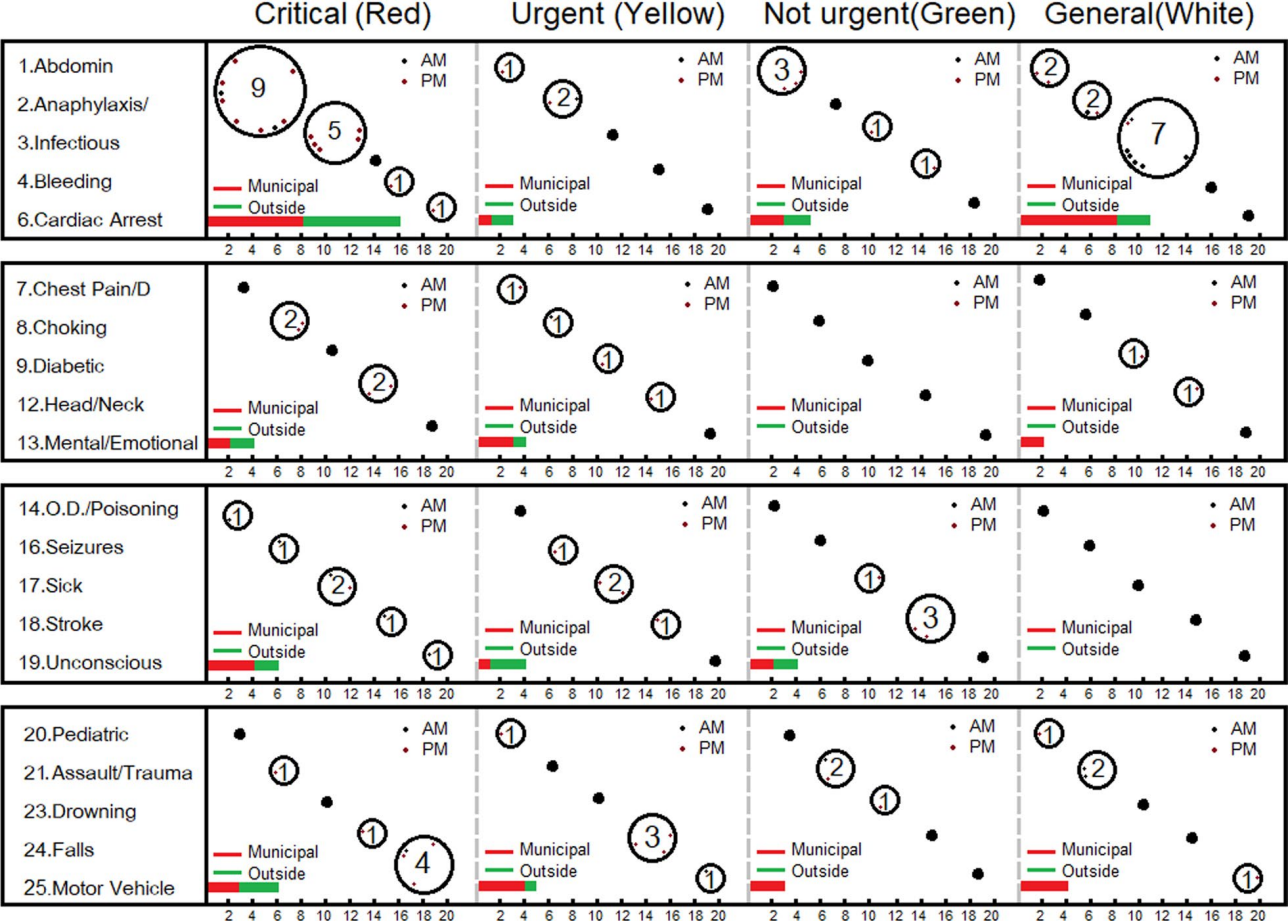
Data retrieved from the cloud database during September–October 2019 after discarding duplicate and missing information. The numbers in the circles represent the frequency with which cases occur, while the dots in the circles indicate the time they occur, separated into AM and PM. Codes 5, 10, 11, 15 and 22 show that no symptoms were reported in the system in that period

After the application had been released to all users in Google Play Store, the data was collected during an 8-week period from September to October 2019. A total number of 128 transactions were found. Missing data, such as a duplicate IDC from the same user and no geolocation information was screened and removed. As a result, a total of 78 transactions of complete IDC commands was analysed in terms of IDC colours, symptoms code, time occurred, and area (Fig. 6).

Figure 6 contains 5 parameters of the analysis. Parameter 1 is a list of code symptoms from 1–25 based on Table 2. Parameter 2 is the essential responses separated by their urgent status based on Table 1. Parameter 3 is the number of cases, which is indicated in a bubble plot. Parameter 4 indicates the time the case was found (like a clock) shown in a bubble plot. Parameter 5 is a comparison of the proportion of municipal and outside-municipal cases.

In terms of the connection between the new patient triage system and the Triage application, it was found in the majority of cases from the real collection that the IDC was red, which indicated critical emergency patients and nine of the cases presented with abdominal/back/groin pain. Most of those with an urgent status were found to be due to Falls/Accidents/Pains, which were reported in the afternoons, but strokes and abdominal/back/groin pain conditions reported during the afternoons were classified as being non-urgent three times. General requests for IDC in the morning were found to be seven times those for infectious diseases. Lastly, users’ locations retrieved from the IDC report showed that cases in urban areas were more frequent than in the countryside. However, most of the critical and urgent cases involved users who lived outside urban areas, while those who lived in towns and cities were classified as non-urgent and general cases. A black IDC indicating no patient was not requested in this study.

The data from an IDC report is important for emergency resources such as ambulances, medical staff or rescue teams to offer support in a timely manner to each patient who is classified as an emergency in each area at times when there is a high number of cases. Hence, the data management function can provide useful information to enhance the performance of the pre-hospital process, which not only benefits patients, but also the operation of rescue teams and medical staff. Based on the traditional protocol, patients had to describe their location in detail when they called the provincial emergency centre or rescue team; in contrast, the geolocation system can correctly detect patients’ location, as confirmed by their feedback. Therefore, the geolocation in this protocol can fully solve the problem encountered by other researchers who applied geolocation to identify the location in emergency cases [26–28]. Although this test did not include an evaluation of the time taken to perform the pre-hospital process, it was confirmed that this function can support and reduce the waiting time or time taken to screen the status of triage patients.

Time is precious in every process, but particularly in emergency cases. The Triagist application provides emergency doctors with information about patients’ status before they arrive at the hospital and the geolocation system in the revised version can help the rescue team to access patients in a timely manner. Therefore, it is clear that the geolocation system in the revised version greatly increases the performance of the emergency operation by reducing the loss of precious time.

## Discussion

The purpose of this research was to extend and revise the Triagist mobile application [14] to support the operation of dispatch centres or rescue teams based on a user-centred design and users’ feedback. Design thinking methodology [33, 42] was used to identify the problems and design a graphical user interface and appropriate functions to support the practical operation of dispatch centres and rescue teams in Thailand. An updated version of the patient triage system was generated based on a review of the literature and an analysis of several emergency medical systems that have been released to support the management of rapid triage healthcare. [12, 13, 16, 29, 30, 32]

Many researchers have proposed methods to increase the efficiency of emergency medical treatment using smart technology. As a result, the present study was focused on developing a system that would enable of patients’ level of emergency to be determined, their need for emergency treatment to be assessed from their IDC results and reveal their geolocation. The system was designed by following the pre-hospital process to increase the efficiency of the EMS [16] based on the concept of CBD to request an IDC [28]. The Laravel framework was used to produce a system that supports a web-based application and cloud base management [37]. The Eight Golden Rules of user interface design and Nielsen’s ten heuristics were employed to design an appropriate practical user interface to support the tasks of emergency staff [35, 36].

The new system designed to support the operation of dispatch centres or rescue teams was connected to the Triagist mobile application, which contains two essential functions. The aim of this research was to resolve the problems currently encountered by emergency staff and increase the performance of the Triagist application to service the primary emergency services in Thailand.

The IDC geolocation function indicates the patient’s condition, location and address, while the IDC management function enables emergency staff to sort cases based on receiving an emergency report. These two functions also support rapid emergency management similar to the current smart medical emergency system, especially the incorporation of geolocation services in the application [27, 29, 31].

Having developed the prototype, a scenario test was conducted in order to ensure that the emergency staff could use the system to make a quick response in the given scenarios. 68 volunteers, including active members of the rescue teams in Chiang Mai and Lampang, as well as emergency medical staff, were recruited to evaluate users’ perception and, hence, achieve another important objective of this study, namely, to design an application that is suitable for users. Therefore, the usability test criteria in this experiment were those applied by Lewis [41] for a possible and rapid evaluation. The results of the evaluation in the usability test were compared between the users in each group, separated by their level of experience and satisfaction. It was found that the group with little experience responded better to the new system than those who were highly experienced. Based on these results, the new system can be used by those with little experience to make a response in practice. This was considered to be the factor that affected the operation of the senior responders and caused them to make a low response [43]. In practice, the senior group were more familiar with traditional methods (manual) than using new technology (mobile). Hence, these results implied that they should be given more information about the purpose of the application, its implementation, instructions of how to use it and suggestions for simple solutions to technical errors in order to increase their confidence in using the app and reduce the barriers to its use [44].

The validated Triagist mobile application was uploaded to Google Play and the IDC results were considered in terms of the density of cases. The analysis of this data will be beneficial for medical staff or rescue teams in allocating medical resources and dealing with urgent cases in a timely manner.

The results of the implementation of both the Triagist mobile application and patient triage system for dispatch centres or rescue teams explicitly show the role of a location-based service linked to the dispatch centre or local emergency services in enabling them to prepare the necessary emergency resources in advance and support their administrative tasks. However, this system was not fully operational according to the overview of the patient triage system in Fig. 1; therefore, this research can be regarded as a second step of the development based on the use of smart information technology.

The patient triage system developed in this study can be regarded as a tool to support dispatch centres, rescue teams or local emergency services. It also reduces the problems currently involved in applying smart health systems in Thailand [23–25]. This is a new approach to the use of smart health systems, especially in the emergency services in Thailand. It can support every user who owns a smart phone, although government policy and other limitations make it more difficult for provincial dispatch centres to use a smart system than rescue teams or local emergency services.

Encouraging the use of the application in the first phase may suitably subsidise the work of rescue teams or local emergency services. This operational system is a low-cost option rather than a system from an overseas country that rescue teams or local emergency services can hire for development purposes. The fact that at least seven rescue teams in northern and southern Thailand have agreed to use the patient triage system in their centres based on this prototype is an indication of the initial success of applying smart technology to emergency services in Thailand; therefore, the results of the present work may help these teams to continue to improve in the future. It is hoped that this project may be a role model to design future functions to improve the flow of emergency responses in Thailand’s pre-hospital process.

## Conclusion

An extended development of Triagist, a mobile application that supports the state of triage management and the operation of dispatch centres or rescue teams has been presented in this paper. The Triagist mobile application was connected to the new patient triage system on a web-based platform that serves the current emergency services in Thailand. One of the contributions of this work is the IDC geolocation function, which identifies patients’ condition, personal information and location. The IDC management function, which provides useful information to support emergency medical management is another contribution. The existence of these functions enables EMS to identify the location of the emergency, leading to the proficient management of the necessary information to make a quick response. All things considered, the extended version of the Triagist mobile application is a crucial tool to enhance the pre-hospital process and especially reduce the loss of precious time for the patient.Besides, this research outcome provides a role model for a low-cost system that dispatch centres or rescue teams can develop by hiring local developers. Finally, the system jumps to the Technology Readiness Level (TRL3), but needs to be extended to the upper level to be developed using innovative technology.

## Limitations

Since the data for this study was collected over an eight-week period and the application was released within the same timeframe, the data obtained cannot cover all the various problems, as well as the different areas. It was difficult to identify all the barriers to the application’s performance in a real life setting due to the short time allocated to collecting the data. In addition, although emergency doctors, dispatch centre staff and rescue teams were initially involved in the design process, the evaluation by external users who do not work at a dispatch centre may lead to the mobile application not being easy to use by future healthcare professionals or other persons who are unfamiliar with mobile apps. Furthermore, the evaluation of the IDC results were not checked by reports from the government’s EMS service that tracks patient transfers. Consequently, the Triagist mobile application requires further development and testing based on the consideration of cost management, resources, responsible assessment, etc.

## Data Availability

The datasets used and analysed during the current study are available from the corresponding author on a reasonable request. The data is not publicly available due to privacy and/or ethical restrictions. The statistical analysis was computed by R programme version 3.1.2

## Abbreviations

AAM: Anglo-American Model
ALS: Advanced Life Support unit
BLS: Basic Life Support Unit
CBD: Criteria-based Dispatch
CSV: Comma Separated Value (in Excel)
ESI: Emergency Severity Index
EMS: Emergency Medical Services
GPS: Global Positioning System
IDC: Initial Dispatch Code
LAN: Local Area Network
LBS: Location-based system
NIEM: National Institute for Emergency Medicine of Thailand
SMS: Short message service
TRL: Technology Readiness Level
CSUQ: Computer System Usability Questionnaire
